# Comparing distress of mouse models for liver damage

**DOI:** 10.1038/s41598-020-76391-w

**Published:** 2020-11-13

**Authors:** Guanglin Tang, Nico Seume, Christine Häger, Simone Kumstel, Kerstin Abshagen, André Bleich, Brigitte Vollmar, Steven R. Talbot, Xianbin Zhang, Dietmar Zechner

**Affiliations:** 1 Rudolf-Zenker, Institute of Experimental Surgery, Rostock University Medical Center, Rostock, Germany; 2 Institute for Laboratory Animal Science, Hannover Medical School, Hanover, Germany

**Keywords:** Experimental models of disease, Gastrointestinal models

## Abstract

In order to foster animal welfare as well as high quality of research, many countries regulate by law that the severity of animal experiments must be evaluated and considered when performing biomedical research. It is well accepted that multiple parameters rather than a single readout parameter should be applied to describe animal distress or suffering. However, since the performance of readout parameters for animal distress is rarely defined and methods for multivariate analysis have only in rare cases been used, it is not known which methodology is most appropriate to define animal distress. This study used receiver operating characteristic curve analysis to quantify the performance of burrowing activity, body weight change and a distress score of mice after induction of liver damage by bile duct ligation or carbon tetrachloride. In addition, Support Vector Machine classification was used to compare the distress of these mouse models. This approach demonstrated that bile duct ligation causes much more distress than carbon tetrachloride-induced liver damage. This study, therefore, provides a prototype how to compare two animal models by considering several readout parameters. In the future these or similar methods for multivariate analysis will be necessary, when assessing and comparing the severity of animal models.

## Introduction

Public discussions on animal welfare have caused the implementation of laws and guidelines to regulate experiments on animals in most countries^1,2^. This made animal welfare a top priority when conducting and publishing in vivo studies^3–5^. Thus, when pursuing animal experiments, scientists have to balance two goals: animal welfare and the potential benefit of research. While this objective is self-evident and coherent, a detailed concept what needs to be done to balance both goals is more difficult to define. In many countries a prospective and often also actual severity assessment of animal experiments are legally required^6^. This should provide the basis for an ethical evaluation and the conclusion, if an animal experiment is justified and, therefore, should be allowed to be conducted.

Thus, an evidence-based analysis of animal distress is often legally required and is also essential for a realistic harm/benefit analysis, a sensible selection of an animal model and the development of refinement strategies. Scientists have primarily used non-invasive methods to assess animal distress. For example, many distress scores based on appearance, behaviour and physical parameters of rodents have been developed^7–9^. In addition, natural behaviour of animals such as burrowing activity has been explored to assess distress^10–12^. One of the most popular parameters to evaluate suffering from animals is body weight which has the distinct advantage that it can be easily and objectively measured^7,13–15^.

While many distinct readout parameters for measuring distress are available, very little is known about how these methods can be compared. The performance of a method or a diagnostic test is usually evaluated by receiver operating characteristic (ROC) curve analysis. The area under the curve (AUC) quantifies this performance and indicates how accurately a test discriminates between two states, typically referred to as diseased and non-diseased state^16^. However, it is well accepted that multiple parameters rather than a single readout parameter should be applied to describe and compare animal distress^7,17,18^. Many studies indeed evaluate several readout parameters for distress, but do not combine these parameters by a statistical procedure to reach a holistic conclusion^13,19–22^. To facilitate such an integrated conclusion, a multivariate analysis, which combines different readout parameters when analysing animal distress, is necessary. Such analyses are often performed in clinical situations in form of a binary logistic regression in order to test whether a combination of biomarkers has higher discriminatory power to differentiate between diseased and non-diseased states than single biomarkers^23,24^. Another option to analyse more than one readout parameter simultaneously is clustering, followed by Support Vector Machine (SVM) classification. For example, clustering was used to differentiate between subgroups of patients with irritable bowel syndrome^25^ or to compare distinct distress levels of mice during colitis^15^.

Thus, it was one aim of this study to evaluate, if ROC curve analysis and binary logistic regression be used to describe the performance of single or multiple readout parameters for defining distress in animals. Moreover, it was the aim to assess whether SVM classification can be used to compare the severity of two animal models. We compared distress caused by bile duct ligation (BDL) to distress caused by carbon tetrachloride (CCl_4_). These two animal models are widely used for studying liver damage and fibrosis^26–30^.

## Results

### Characterisation of parameters measuring distress after BDL

**Figure 1:**
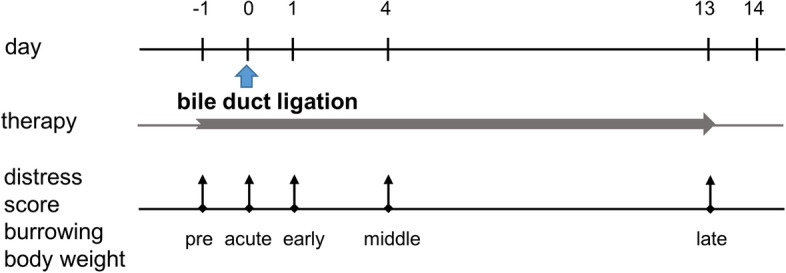
Scheme describing how the experiments for the bile duct ligation (BDL) animal model were performed. Surgery was done on day 0 and the distress parameters were evaluated during pre, acute, early, middle and late phase on the indicated days. Therapies with MCC950 or vehicle solution were performed by daily subcutaneous injection from day − 1 to day 13.

Mice were evaluated before and after BDL during the early, middle and late phases of cholestasis by assessing a distress score, burrowing activity and body weight (Fig. 1). First of all, we aspired to evaluate the suitability of these parameters to measure distress of mice. We hypothesized that parameters, which are suitable to measure distress should be able to differentiate between healthy and diseased mice as well as between mice which survived and non-survivors.

**Figure 2:**
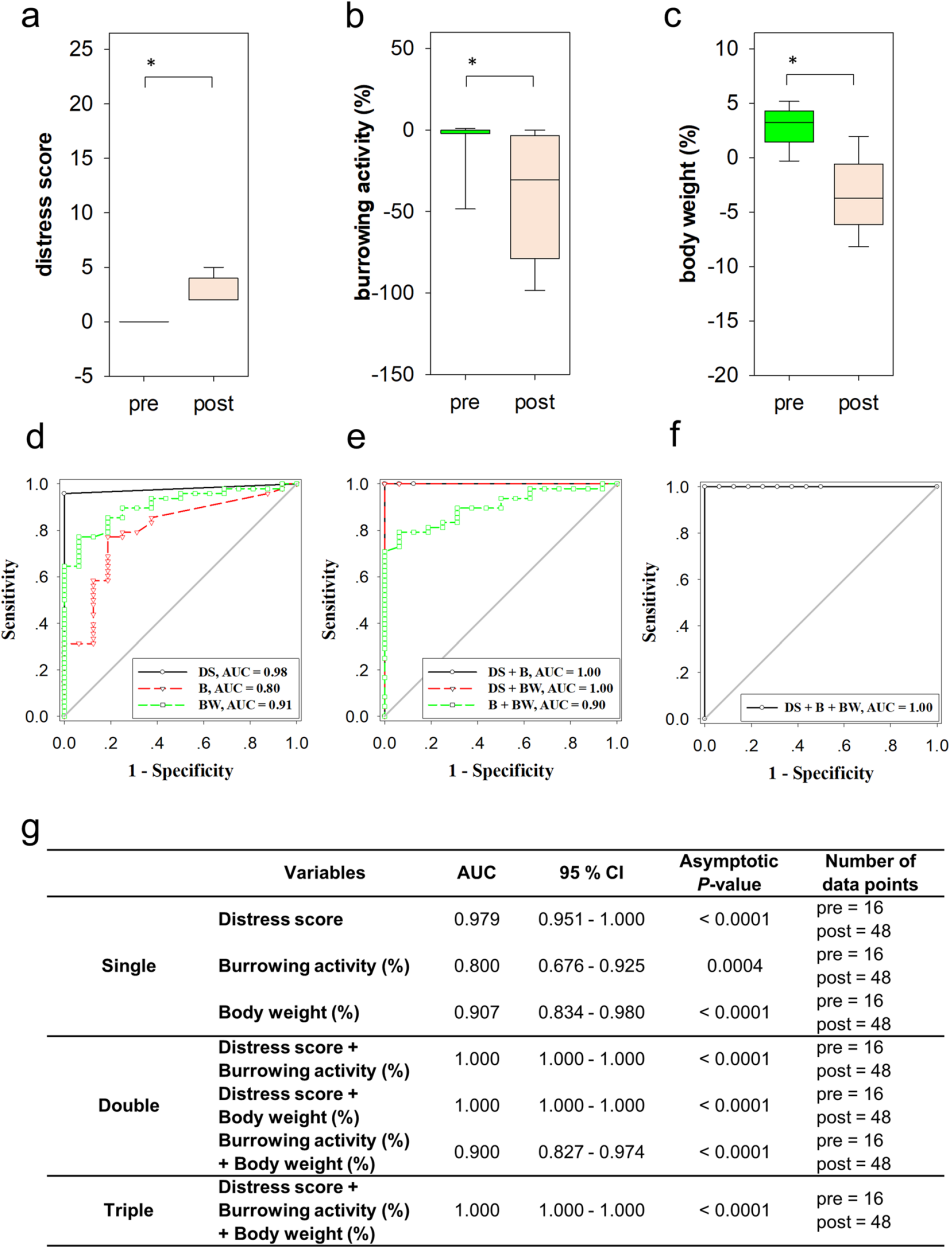
Distress before and after BDL. The distress score (**a**) was increased (Mann–Whitney rank sum test, *P* ≤ 0.001), burrowing activity (**b**) was decreased (Mann–Whitney rank sum test, *P* ≤ 0.001) and body weight (**c**) was also decreased (Mann–Whitney rank sum test, *P* ≤ 0.001), when comparing data taken before BDL (pre) to data taken after BDL (post). ROC curve analysis that computed the area under the curve (AUC) for single (**d**), two (**e**) or all three (**f**) distress parameters. The performance of single and multiple parameters is described by presenting the AUC, the 95% confidence interval (CI) and the asymptotic P-value (**g**). Data of 16 mice, pre: n = 16 data points, post: n = 48 data points.

Thus, we first analysed mice, which survived until day 14 (survivors), in order to explore, if these read out parameters could differentiate between healthy and diseased mice. While the distress score increased continuously after BDL, the burrowing activity and body weight of mice rather decreased after this intervention (supplementary Fig. S1). No significant change in any of these parameters was observed when treating the mice with the NLRP3 inflammasome inhibitor MCC950 (supplementary Fig. S1), although previous studies suggested that this inhibitor can have analgesic function^31^. Thus, all BDL cohort mice were pooled and distress before BDL (pre) was compared to distress after BDL (post). We observed that BDL led to a significant increase of the distress score (Fig. 2a). It caused a significant decrease of burrowing activity (Fig. 2b) and a reduction of body weight (Fig. 2c). This suggests that distress score, burrowing activity and change in body weight are sensitive parameters that can differentiate between distress before (level 0) and after BDL (level 1). To evaluate the performance of these parameters in distinguishing between these two distress levels, we used ROC curves. We observed that all parameters, distress score, burrowing activity and body weight, can discriminate between these two distress levels (Fig. 2d). Combining multiple distress parameters with binary logistic regression revealed that the combination of distress score plus burrowing activity, distress score plus body weight and the combination of all three parameters produced a very high AUC indicating a very good performance in defining distress (Fig. 2e–g).

We also evaluated, if distress parameters could differentiate between different magnitudes of cholestasis. ALP activity has been demonstrated to increase with the progression of cholestasis^32^. Therefore, we evaluated ALP activity of mice after 2, 5 or 14 days of cholestasis and used k-means clustering to discretize the data into two categories: Low ALP and high ALP. Surprisingly, we observed that neither the distress score nor the burrowing activity could differentiate between low ALP and high ALP animals (supplementary Fig. S2). However, body weight change could differentiate well (AUC = 0.79) between these two clusters (supplementary Fig. S2). In order to analyse, if other parameters measuring distress would improve the differentiation between low ALP and high ALP animals, we determined the corticosterone concentration in the blood plasma (supplementary Fig. S2). Indeed, the corticosterone concentration in the blood plasma of animals could also differentiate well (AUC = 0.72) between low and high ALP animals (supplementary Fig. S2). However, when combining body weight change and corticosterone concentration in a logistic regression the discriminatory power of the combination was not higher than the discriminatory power of only the body weight change (supplementary Fig. S2). Thus, for differentiating between low and high ALP animals analysing body weight change is sufficient. Possibly, a combination with yet unknown additional distress parameters might be needed to predict the magnitude of cholestasis with an even higher discriminatory power.

**Figure 3:**
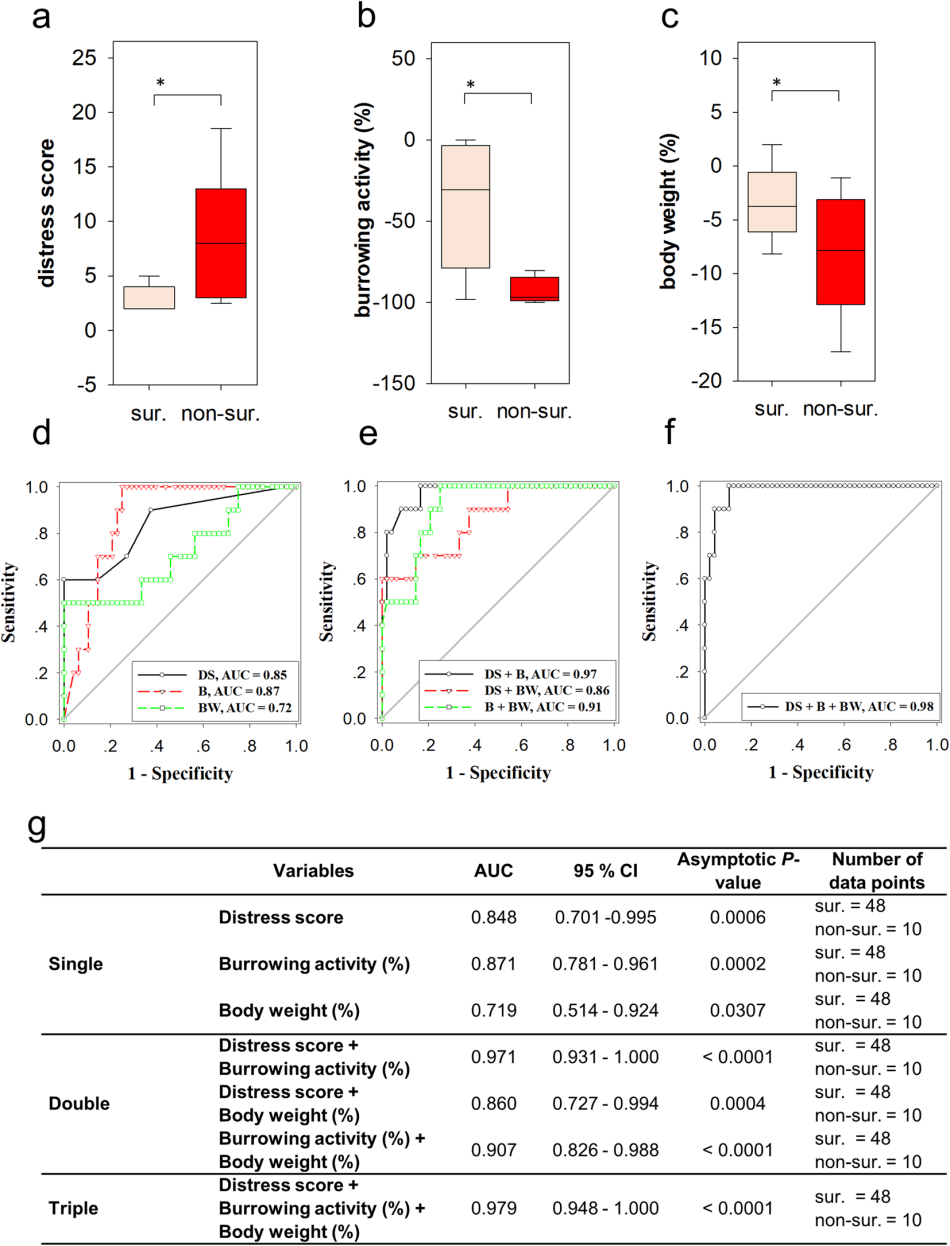
Distress of survivors and non-survivors after BDL. The distress score (**a**) was increased (Mann–Whitney rank sum test, *P* ≤ 0.001), whereas burrowing activity (**b**) was decreased (Mann–Whitney rank sum test,* P* ≤ 0.001) and body weight (**c**) was also reduced (Mann–Whitney rank sum test, *P* = 0.031), when comparing data of survivors (sur.) to data of non-survivors (non-sur.). ROC curve analysis shows the area under the curve (AUC) for single (**d**), two (**e**) or all three (**f**) distress parameters. The performance of single and multiple parameters is described by presenting the AUC, the 95% confidence interval (CI) and the asymptotic P-value (**g**). Survivors: 16 mice, 48 data points; non-survivors: 10 mice, 10 data points.

We then explored, if mice which did not survive until day 14 (non-survivors) reached a different distress level before death when compared to mice that survived after BDL. We observed that the distress score of non-survivors measured before death is significantly higher than the distress score of survivors (Fig. 3a). The burrowing activity (Fig. 3b) and body weight (Fig. 3c) of non-survivors were significantly lower than those of surviving mice. These data suggest that non-survivors experience increased distress before death (level 2) when compared to surviving mice (level 1). In order to evaluate the performance of the readout parameters in distinguishing between these two distress levels, we used ROC curves. All single readout parameters such as distress score, burrowing activity and change in body weight had discriminatory power to differentiate between survivors and non-survivors (Fig. 3d). After combining multiple distress parameters with binary logistic regression, we observed that combination of two or three parameters also had a high discriminatory power (Fig. 3e,f). The combination of all three parameters (distress score plus burrowing activity plus body weight) produced the largest AUC, suggesting that the combination of all readout parameters allows the best differentiation between survivors and non-survivors (Fig. 3g). These data, therefore, suggest that the distress score, burrowing activity and body weight are suitable parameters to describe distinct distress levels.

### Considering multiple parameters when differentiating between two distress levels

**Figure 4:**
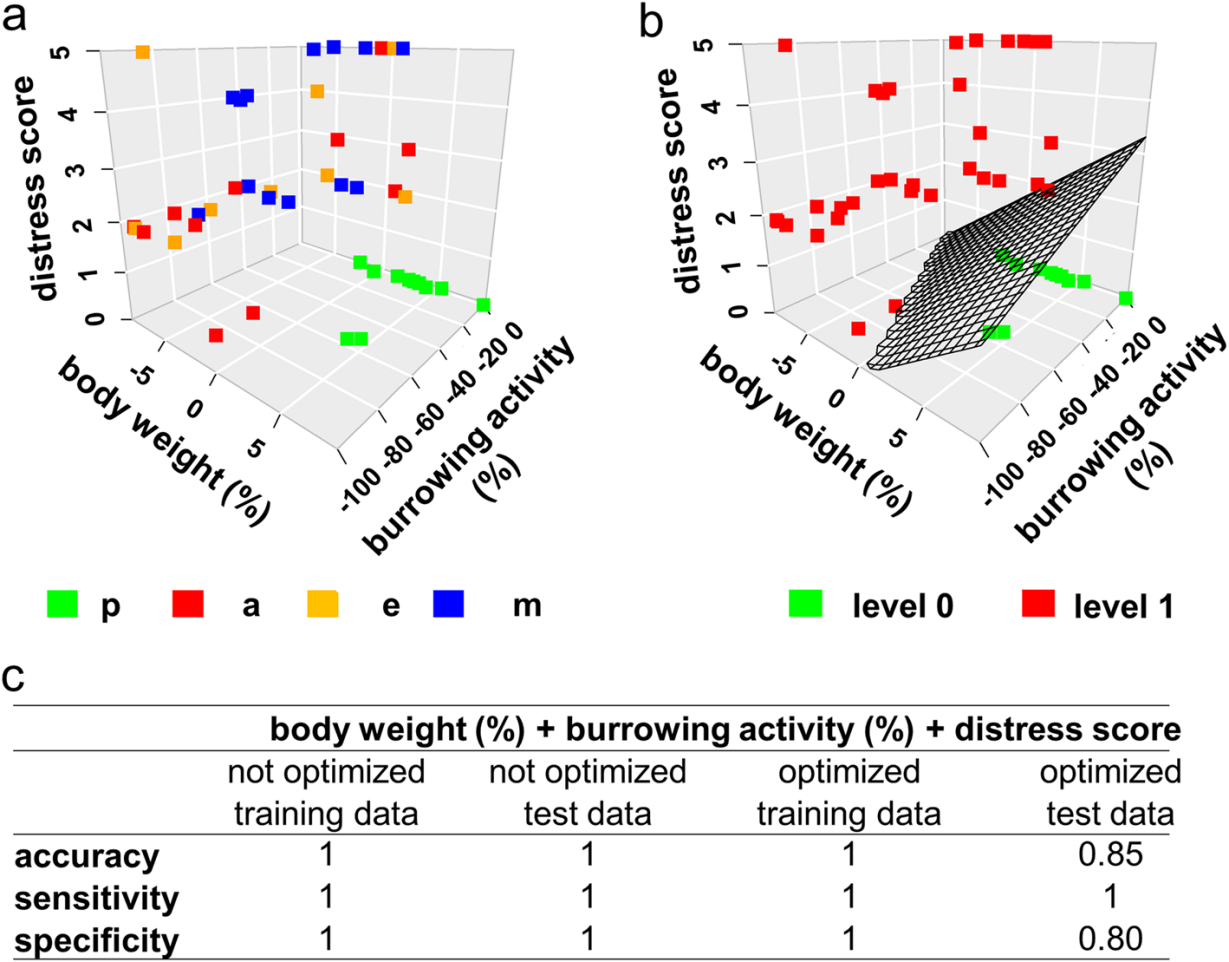
Generation of a training model by SVM. Single data points (squares), which were derived from the training data set from identical animal previous (p) to BDL and at the acute (a), early (e), and middle (m) phase of cholestasis are presented in form of a three dimensional scatter plot (**a**). A discriminatory model was built by training a linear SVM kernel to the labelled data in order to differentiate between two levels (level 0 and level 1) of distress (**b**): The resulting classifier (hyperplane) discriminates between these two levels. The accuracy, sensitivity and specificity of the training model was characterised using either the training data themselves or a test data set and applying the hyperplane (not optimized) or an optimized hyperplane after a 5-times repeated tenfold cross validation (**c**). Training data set: n = 11 data points (pre), post: n = 33 data points (post); test data set: n = 5 data points (pre), post: n = 15 data points (post).

Next, we evaluated whether all three parameters can be used together to discriminate between the distress of healthy (pre-intervention) against the distress of diseased animals (post-intervention). We used machine learning to address this question: more specifically, we used a Support Vector Machine (SVM) to classify samples. Class-labels were obtained by labelling pre- against post-intervention data. For subsequent classification, we first split the data randomly into a training (containing 70% of data) and a test data set (containing 30% of data). The model was then built using the training data (Fig. 4a). Within the SVM, a linear kernel function was used to find the classifier. This tuned and optimized discriminator was visualized in the plots as a hyperplane, separating two putative levels of distress, which were defined as distress level 0 or distress level 1 (Fig. 4b).

For internal model optimization, and to address potential sampling bias we used hyper-parameter tuning and fivefold repeated tenfold cross-validation. The mean accuracies, sensitivities and specificities from this process were reported for the model (Fig. 4c shows results for both, the optimized and non-optimized model). The model itself was validated using the excluded (and labelled) test data (Fig. 4c). We observed high accuracy, sensitivity, and specificity for training as well as test data (Fig. 4c). This suggests that the combination of all three parameters (distress score, burrowing activity, bodyweight) exhibits a high diagnostic ability for the differentiation between distress level 0 and distress level 1. The rigorous model design and cross-validation process further ensured that these results are not based on potential sampling bias. Also, the optimized model shows lower accuracies for the external test data (accuracy optimized model: 0.80; accuracy not optimized model: 1). This was expected as the not-optimized models tend to overfit the data.

### *Comparing distress of the BDL to the CCl*_*4*_* animal model*

**Figure 5:**
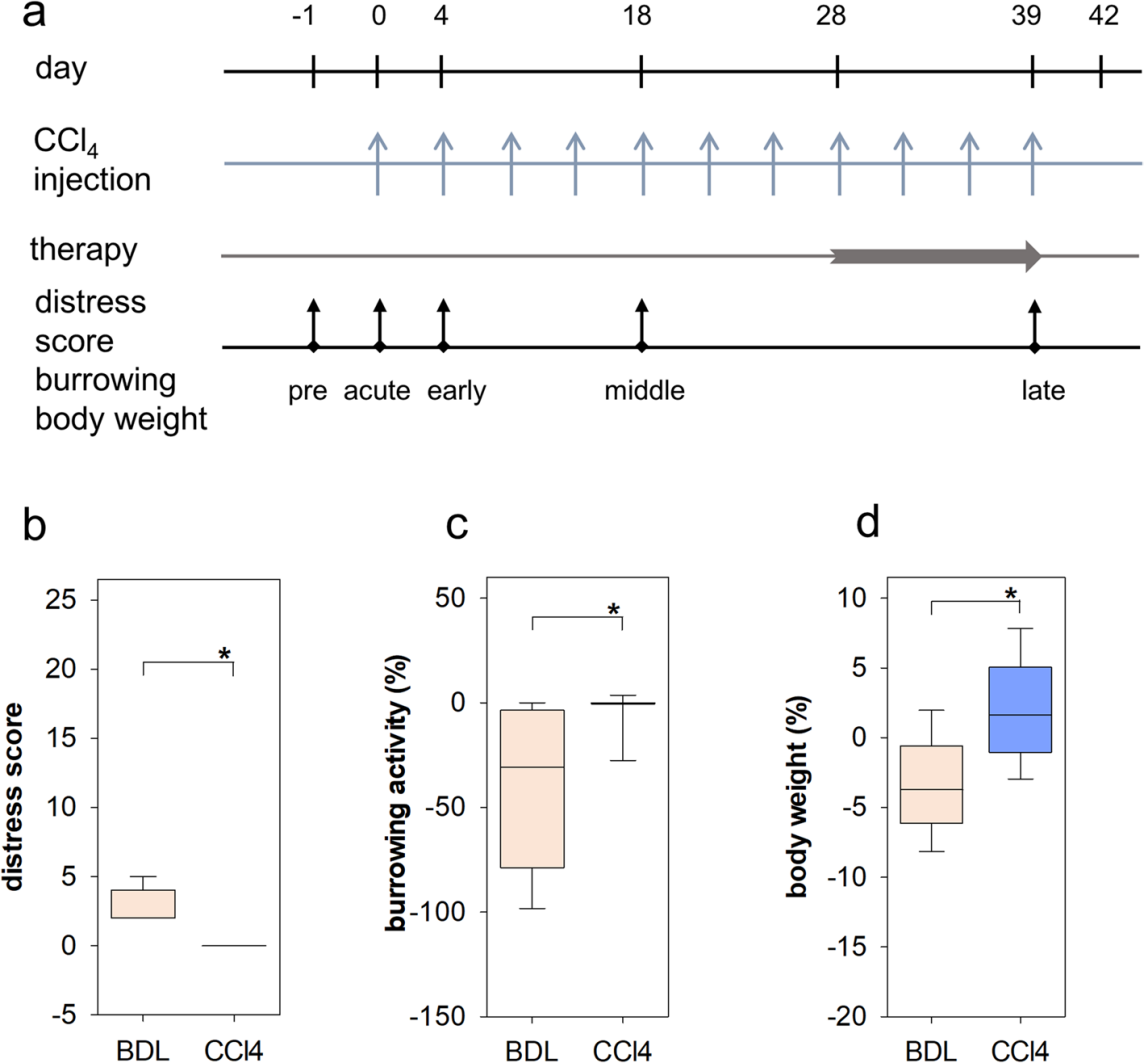
Distress of mice after CCl_4_ injections and after bile duct ligation. Scheme describing how the experiments for the CCl_4_ animal model were performed (**a**): CCl_4_ was injected sc on the indicated days, the distress parameters were evaluated during pre, acute, early, middle and late phase and a therapy with MCC950 or vehicle solution was performed as daily subcutaneous injections from day 28 to 39. The distress score (**b**) was decreased (Mann–Whitney rank sum test, *P* ≤ 0.001), whereas burrowing activity (**c**) was increased (Mann–Whitney rank sum test,* P* ≤ 0.001) and body weight loss (**d**) was decreased (Student’s t test,* P* ≤ 0.001) when comparing post-BDL to post-CCl_4_ animals. BDL: 16 mice = 48 data points; CCl_4_: 10 mice = 30 data points.

Next we pursued the question if and how we can compare the distress between two animal models. In order to compare the BDL model to another animal model widely used for studying liver damage and fibrosis, mice were repetitively injected with CCl_4_ (Fig. 5a). These mice were also either treated with MCC950 or a vehicle control and the distress of these animals was analysed before any intervention and during the early, middle and late phases of disease progression by assessing the distress score, burrowing activity and body weight (Fig. 5a). Again, no significant change in distress score, burrowing activity and body weight was observed when treating the mice with MCC950 or a vehicle control (data not shown). Thus, all CCl_4_ cohort mice were pooled and post-CCl_4_ and post-BDL data were then compared (Fig. 5b–d). We observed that CCl_4_-treated mice had a significantly decreased distress score (Fig. 5b), increased burrowing activity (Fig. 5c) and significantly less body weight reduction (Fig. 5d), when compared to BDL mice. Thus, all three read out parameters indicate that CCl_4_ causes less distress than BDL.

**Figure 6:**
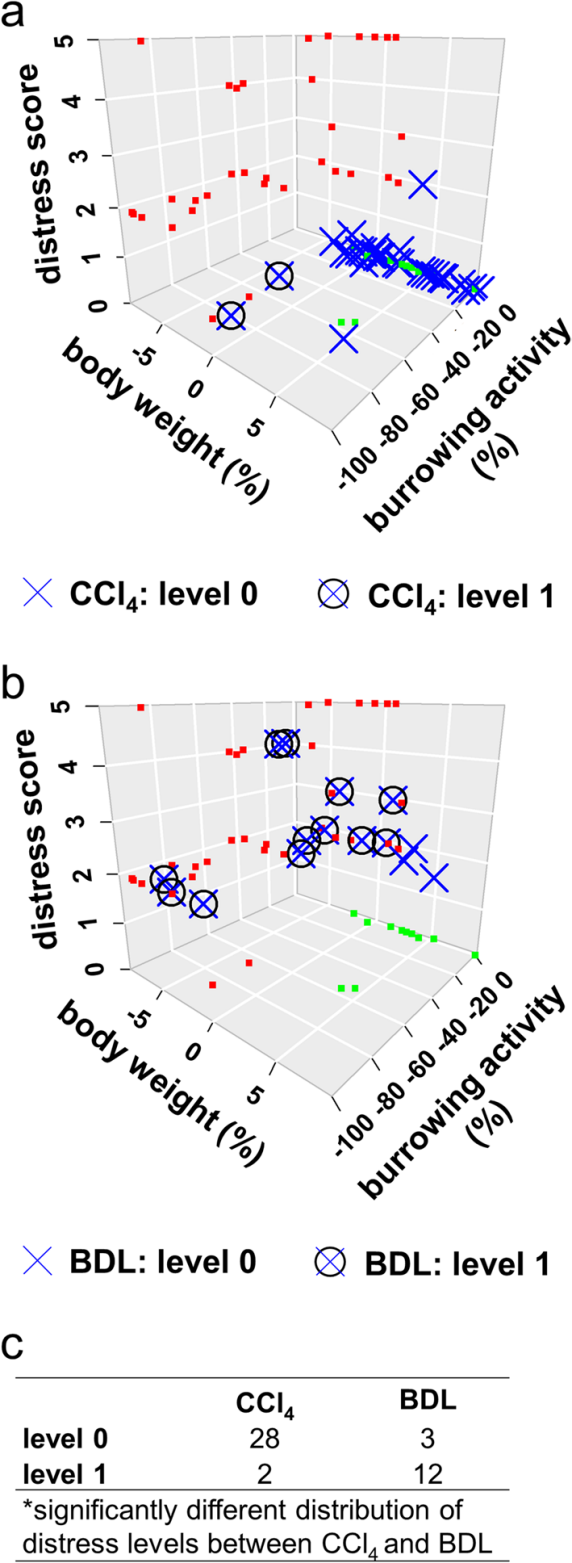
Comparing distress caused by CCl_4_ injections or bile duct ligation using SVM classification. In both plots green and red squares indicate distress level 0 or distress level 1 of the BDL training data set and crosses denote data classified as distress level 0, whereas circled crosses denote data classified as distress level 1. Blue crosses denote post-CCl_4_ (**a**) and post-BDL (**b**) distress. A 2 × 2 contingency table compares the distributions of predicted distress levels of post-CCl_4_ to the post-BDL test data set (**c**). A significantly different distribution of distress levels between these data sets has been determined by Fisher’s Exact Test, *P ≤ 0.001), CCl_4_: n = 30 data points; BDL: n = 15 data points.

We then compared these two animal models by using the optimized training model based on 70% of the BDL data. We then classified the post-CCl_4_ data according to this training model (see blue crosses in Fig. 6a). In addition, we classified the post-BDL data of the test data set (see blue crosses in Fig. 6b). Only 2 out of 30 post-CCl_4_ data points were assigned to distress level 1, whereas 12 out of 15 post-BDL data points were correctly assigned to distress level 1 (Fig. 6c). Using Fisher’s exact test, a significant difference in the distress levels distribution between BDL and the CCl_4_ cohort was observed (*P* < 0.001). This multivariate analysis suggests that at most time points CCl_4_-treated animals experience less distress than animals after BDL.

In order to compare liver damage in both animal models, we assessed the activity of aspartate aminotransferase (AST), alanine aminotransferase (ALT) and glutamate dehydrogenase (GLDH) in blood plasma. AST and ALT activity was significantly increased in cholestatic as well as CCl_4_-treated mice, when compared to heathy control animals (supplementary Figure S3). GLDH was significantly increased in cholestatic animals when compared to healthy or CCl_4_-treated mice (supplementary Figure S3). In addition, we also evaluated oxidative stress by measuring malondialdehyde in liver tissue. Malondialdehyde was significantly increased after repetitive CCl_4_-treatment when compared to cholestatic or healthy mice (supplementary Figure S3). These results demonstrate that the liver is damaged after cholestasis and toxic liver injury, but that specific pathophysiological features such as the induction of oxidative stress differs between these two animal models.

## Discussion

There is an urgent need to evaluate the feasibility of methods to compare distress caused by different animal models^33^. The present study compared BDL to CCl_4_-induced liver damage and evaluated animal distress based on three distinct readout parameters. The multivariate analysis using SVM clearly demonstrated that BDL caused more distress than the treatment with CCl_4_.

No direct multivariate comparison of distress between BDL and CCl_4_-induced liver damage has been published to our knowledge. However, publications describe an average body weight loss of 15–20%, 18%, or 20–30% after BDL^34–36^ or a transient body weight loss of approximately 8% or 10% during repetitive CCl_4_ injection^37,38^. This supports our conclusion that BDL causes more distress than CCl_4_. However, the BDL animal model will still be needed for the following reasons. Distinct animal models are necessary to address the central principle of science that robust research needs many independent lines of evidence^39^. Indeed, BDL and CCl_4_-induced liver damage are often used in one study to prove a scientific conclusion in two independent animal models^40,41^. In addition, there are also some differences between these two animal models. BDL causes an increase in biliary pressure, inflammation and cytokine secretion resulting in proliferation of biliary epithelial cells and portal fibrosis^42^. BDL therefore mimics cholestatic injury, which is, for example, observed during autoimmune diseases (primary biliary cirrhosis and primary sclerosing cirrhosis) and obstructive conditions such as cholelithiasis and tumour compression of bile ducts^43^. In contrast, metabolites of CCl_4_, such as trichloromethyl radicals, induce oxidative stress, centrilobular liver necrosis, an inflammatory response and liver fibrosis^42,44^. In many aspects, it mimics liver damage in humans by different toxins^42^. These distinct pathophysiological features and mechanisms of animal models will remain to be of utterly importance, when deciding which animal model will be used for addressing a specific scientific hypothesis. However, at least for the BDL animal model, the use of analgesics should be essential^4^. It is especially necessary to mention this point, if one considers that only 3.4% of studies, which describe experiments using BDL in mice, specified the administration of a systemic analgesic^45^. This is surprising, considering that it was already demonstrated decades ago that animals experience post-operative pain after BDL^46^. However, analgesia can also interfere with disease mechanisms and can actually be harmful to animals when applied in high doses^47,48^.

The most important prerequisite for being able to judge animal distress are methods with high discriminatory power to differentiate between distinct distress levels. This study used ROC curve analysis to evaluate the discriminatory power of readout parameters. This tool has been widely used to define the diagnostic ability of methods in a clinical situation. For example, ROC curve analysis helped to define which biomarker in the blood has the best discriminatory power to predict pancreatic cancer^23^ or which biochemical marker is suitable to predict increased risk of stillbirth in women with intrahepatic cholestasis of pregnancy^49^. In our study ROC curve analysis judged the suitability of readout parameters to differentiate between healthy mice and diseased mice or between diseased mice, which survive, and diseased mice, which will succumb to their disease. All readout parameters: distress score, burrowing activity and body weight change had discriminatory power to differentiate between animals before and after induction of cholestasis (Fig. 2d). However, burrowing activity was the parameter with the lowest performance (performance of parameters: distress score > body weight change > burrowing activity). When differentiating between survivors and non-survivors all readout parameters had again a high discriminatory power (Fig. 3d), but body weight change was the parameter with the lowest performance (performance of parameters: burrowing activity > distress score > body weight change). In addition to assessing the discriminatory power, one can determine the optimal cut-off of a diagnostic method by Youden’s index and calculate the positive predictive value (PPV)^50^. We, therefore, also calculated the PPV using the combination of all three parameters. An optimal cut-off calculated by Youden’s index lead to 5 false positive and 10 true positive predictions, resulting in a PPV of 67%. Thus, it is not practical to use this method for deciding, if animals should be euthanized, because one would kill too many animals, which would otherwise survive. However, the combination of all three parameters is useful in describing distinct distress levels and can be used to compare 2 different animal models. These experiments also demonstrate that not a single readout parameter can be used as the gold standard for all situations.

This need for considering multiple parameters to assess animal welfare was often postulated^7,17,18^. However, in many studies several parameters are evaluated, but these parameters are often not combined by a statistical procedure to reach a holistic conclusion^13,19–22^. Only very few studies exist, which use biostatistical methods to combine distinct readout parameters for defining animal distress. For example, Peng et al*.* have used composite z scores to compare the results of several behavioural tests between control mice and mice after surgery^20^. Häger et al. have used k-means clustering to compare distinct distress levels during colitis^15^. Möller et al. have used principal component analysis to describe many behavioural and biochemical variables supporting the conclusion that there is no major difference in distress between rats after electrode implantation and rats after electrode implantation plus kindling of epilepsy^51^. In our study we plotted three parameters and defined distress levels by SVM classification. This method had a high specificity, sensitivity and accuracy when validated with test data (Fig. 4c). However, we also want to emphasize that ROC curve analysis indicated that single read out parameters or two read out parameters, which were combined by multiple logistic regression, have also a very high discriminatory power to differentiate between distress levels in the BDL animal model (Fig. 2g). This indicates that less than three readout parameters might suffice to define the distress of animals and to compare animal models. However, we propose that substantiating a conclusion by considering several readout parameter is better to than relying on only one single parameter. Such a multivariate conclusion reduces arbitrariness when choosing a readout parameter and therefore diminishes bias when comparing animal models.

Although this publication suggests that SVMs can be used to compare the distress of two animal models, it is premature to claim that this method will allow us to determine the severity of all animal models in a scientific and rational manner. First, distinct research facilities will have to test if this or similar methods can be applied to many different animal models to compare distress between distinct models. Second, accessible tools to assess and compare distress have to be provided for the scientific community. Talbot and colleagues have started to explore such a tool, and recommend the use of a Relative Severity Assessment (RELSA) score for comparing animal models^52^. It will be important for the research community to make such tools accessible online. Third, the scientific community will have to provide a network of comparing distress between the most essential animal models. Only if this network allows an arrangement of animal models according to their distress level, one could start grading evidence-based severity into categories (e.g. mild, moderate or severe) as demanded by the legislation of many countries.

## Methods

### Animals

This study was conducted in accordance with the European directive 2010/63/EU and national law. All experiments were approved by the local ethics committee of the public authority (Landesamt für Landwirtschaft, Lebensmittelsicherheit und Fischerei Mecklenburg-Vorpommern, 7221.3-1-002/17). Because female mice were used to expand the mouse strain, surplus male BALB/cANCrl mice were used for this study. Please note that the focus on male mice might be a limitation of this study. A few mice of this mouse strain were purchased from Charles River (Wilmington, MA USA) and bred in the central animal facility of the Rostock University Medical Center (the health of the animal stock is routinely checked according to FELASA guidelines). Before the experiment the mice had more than 2 days for acclimatization. Animals were allocated in a non-random manner matching the age of both treatment groups and the experimenters were not blinded when injecting drugs. Distress was evaluated by two people (GT, NS), and in case of difficulties, in addition by another person (DZ). The required number of animals was calculated before starting the experiments by sample size calculation (alpha = 0.05, power = 0.8). Mice were group housed during breeding and the first few days before the actual experiments. Afterwards they were single housed in Eurostandard Type III clear plastic cages with wire lid, light/dark cycle of 12 h/12 h (dawn: 6:30–7:00 am) at a temperature of 21 ± 2 °C, with a relative humidity of 60 ± 20%. Autoclaved bedding (Bedding Espe Max 3–5 mm granulate, H 0234-500, Abedd, Vienna, Austria), shredded tissue paper (PZN03058052, FSMED Verbandmittel GmbH, Frankenberg, Deutschland), one paper tunnel (75 × 38 mm, H 0528-151, ssniff) and a wooden enrichment tool (Espe size S, 40 × 16 × 10 mm), H0234.NSG, Abedd). Food (pellets, V1534.000, 10 mm, ssniff) and tap water ad libitum were provided. Mice were euthanized by quickly anaesthetizing them with 5 vol % isoflurane and killing them with cervical dislocation.

### Induction of liver damage

For inducing cholestasis by BDL on day 0, mice were quickly anaesthetized by 5 vol % isoflurane (CP-pharma, Burgdorf, Germany) and placed on a heating plate (37 °C). Then the laparotomy was performed under anesthesia (1.2–2.5 vol % isoflurane). As described in a previous study^53^, the common bile duct was ligated by three surgical knots and was then transected between the two distal ligations. After closing the abdominal cavity, each mouse was allowed to recover from anesthesia in a single cage in front of a red warming lamp. The surgical procedure took 25–40 min. To relieve pain, 5 mg/kg carprofen (Pfizer GmbH, Berlin, Germany) was injected (sc) before operation and 0.25 ml metamizol (500 mg/ml, Ratiopharm GmbH, Ulm, Germany) was added to the drinking water (100 ml, drinking water was changed daily) until euthanasia of the mice. Supportive care was given after BDL by offering soaked food to all animals until euthanasia. In order to evaluate, if the NLRP3 inflammasome inhibitor MCC950 (Sigma Aldrich, St. Louis, USA, code PZ0280) could impair distress, 20 mg/kg MCC950 or aqua dest. ad inj. (Sham) was ip injected daily from day 1 before BDL to day 13 after BDL. For inducing liver damage by CCl_4_ (Merck Millipore, Eschborn, Germany, code 1.02209.1000), this substance was diluted fourfold with corn oil (Sigma-Aldrich, code C8267). Per g body weight 1 µl of this solution (dose of CCl_4_: 0.25 ml/kg body weight) was injected (ip) between 14:40–15:00 into the mice twice per week until day 42 (on day 0, 4, 7, 11, 14, 18, 21, 25, 28, 32, 35, 39). To relieve pain, 0.25 ml metamizol (500 mg/mL, Ratiopharm GmbH, Ulm, Germany) was added to the drinking water (100 ml) until euthanasia of the mice. 20 mg/kg MCC950 or aqua dest. ad inj. (Sham) was injected (ip) daily from day 28 to day 41 after first CCl_4_ injection. The sixteen BDL mice (survivors) were at the beginning of the experiment 10.29/8.07–18.61 (median/interquartile range) weeks old and had 27.11/21.80–29.68 (median/interquartile range) g body weight, whereas ten BDL mice (non-survivors) were at the beginning of the experiment 9.79/8.36–12.20 weeks old and had 24.90/23.83–26.23 g body weight. The ten CCl_4_-treated mice were at the beginning of the experiment 7.86/7.86–8.14 weeks old and had 24.52/22.99–24.97 g body weight.

### Evaluation of animal distress

#### Burrowing

To evaluate burrowing activity of mice, a tube (length: 15 cm, diameter: 6.5 cm) filled with 200 g of food pellets was placed into the cage 2–3 h before the dark phase^54^. The remaining pellets in the burrowing tube were weighed after 17 ± 2 h and the weight of the burrowed pellets was calculated. Burrowing activity was measured before the first intervention (pre) and during the acute (day 0), early (BDL: day 1, CCl_4_: day 4), middle (BDL: day 4, CCl_4_: day 18) and late (BDL: day 13, CCl_4_: day 39) phase of liver damage. The burrowing tube was always placed into the cage 1 ± 0.5 h after CCl_4_ injection. Changes in burrowing activity were calculated by using the weight of burrowed pellets on day 7 before BDL and on day 8 before CCl_4_ injection as a reference for the respective cohort.

#### Distress score

The wellbeing of mice was assessed by evaluating multiple parameters with the help of a distress score^55^. When the total score was higher than 15, the affected mouse was euthanized in order to avoid further deterioration of health. Distress was assessed before the first intervention (pre) and during the acute (day 0), early (BDL: day 1, CCl_4_: day 4), middle (BDL: day 4, CCl_4_: day 18) and late (BDL: day 13, CCl_4_: day 39) phase of liver damage. The distress was always evaluated 30 ± 5 min after CCl_4_ injection.

#### Body weight

The body weight of mice was assessed before the first intervention (pre) and during the acute (day 1), early (BDL: day 2, CCl_4_: day 5), middle (BDL: day 5, CCl_4_: day 19) and late (BDL: day 14, CCl_4_: day 40) phase of liver damage. Thus, in all experiments the body weight was determined 1 day after measuring distress by a score sheet or by burrowing activity. This allows enough time for a body weight adjustment to a specific distress level (e.g. after injection of CCl_4_).

#### Blood plasma and tissue analysis

AST, ALT, GLDH and ALP activity were spectrophotometrically assessed in blood plasma using the Cobas c111 analyser (Roche GmbH, Mannheim, Germany). For determining the corticosterone concentration in blood plasma the mouse and rat ELISA-Kit (DEV9922, Demeditec Diagnostics GmbH, Erfurt, Germany) was used according to the instructions of the manufacturer. Oxidative stress was evaluated by measuring the total malondialdehyde concentration after hydrolysing liver tissue at pH 1–2 and using the BIOXYTEC MDA-586 kit from OxisResearch (OXIS Health Products Inc. Portland, OR, USA).

#### Data presentation and statistical analysis

In line graphs data are presented as mean value ± standard deviation, whereas box plots indicate median interquartile range as well as 90% percentile and 10% percentile in form of whiskers. The characteristics of data were assessed by Shapiro–Wilk normality test and by Levene median equal variance test. Student’s t-test (based on normal distribution and equal variance of data) or the Mann–Whitney Rank Sum test were used to determine the significance of differences. When comparing two groups, differences with *P ≤ *0.05 were considered to be significant. When comparing treatment groups at several time points, differences were only considered to be significant, when the *P*-value was lower than 0.05 divided by the number of meaningful comparisons (Bonferroni correction for multiple comparisons). These evaluations were done using SigmaPlot 12.0 (SYSTAT Software Inc., San Jose, USA; https://systatsoftware.com/products/sigmaplot/). For box plots, ROC curves, logistic regressions and Support Vector Machine classification, data of the pre- and post-intervention phase (all data from the acute, early and middle phase) were used to differentiate between healthy and diseased animals. For differentiating between post-BDL survivors and non-survivors, all data of surviving mice of the acute, early and middle phase after BDL were compared to data measured 0–2 days before death or euthanasia of non-survivors.

ROC curve analysis (using SigmaPlot 12.0, SYSTAT Software Inc.) determined the area under the curve (AUC) with the respecting 95% confidence intervals (CI) as a measurement for the performance of the readout parameters^56^. In addition, this analysis gives the asymptotic *P*-value that determines if the AUC is significantly different from AUC = 0.5. To analyse the efficacy of the combination of two or three parameters, the data sets were combined by binary logistic regression using SigmaPlot 12.0 and the ROC curves were calculated afterwards.

In order to analyse distress considering all three readout parameters simultaneously, a Support Vector Machine was built on a 64-bit computer with 32 GB RAM using the R software^57^ with the following packages: caret^58^ and e1071^59^. Prior to model building, samples were class-labelled using the experimental time phases (pre- vs. post-intervention). Categories were labelled as level 0 (pre) and 1 (post) and used in the classification process. Samples were randomized into 70% training and 30% test data prior to model building. A linear kernel function (u'∙v) was then used to construct the SVM-classifier with the training data. Data were scaled for the building process. The non-optimised fit was then tuned for the hyper-parameter cost function to optimise the SVM margin width for the classifier. In parallel, the tuning process was stratified using fivefold repeated tenfold cross-validation. The mean from all internal validation runs was then used to construct the optimised classifier. Model performance was reported in two stages: (a) re-classification (prediction) of the training data against the model (non-generalizable internal performance check) and (b) classification of the external test data (validation). In each case, data from a confusion matrix (accuracy, sensitivity, specificity) was reported for both, the optimised and the non-optimised model. The resulting values reflect model stability and also compensate for low sample sizes via repeated cross-validation. The externalised test data further assess the generalisability of the model. Finally, the hyperplane was constructed by coefficient extraction and grid extension of the optimised SVM model. When comparing CCl_4_ cohorts to BDL, the optimized model was used to predict severity classes for post-intervention BDL data from the externalized test set as well as post-intervention CCl_4_ data. The predictions were plotted in a scatterplot and class differences analyzed by Fisher’s Exact Test.

## Supplementary information

Supplementary Information

## Data Availability

The authors declare that all data supporting the findings of this study are available within the paper and its supplementary information file.

## References

[1] 1*National Research Council (US) Committee for the Update of the Guide for the Care and Use of Laboratory Animals. Guide for the Care and Use of Laboratory Animals (Eighth Edition)*. (National Academy of Sciences, 2011).

[2] 2Directive 2010/63/EU of the European Parliament and of the Council of 22 September 2010 on the protection of animals used for scientific purposes (Text with EEA relevance). Available from: https://eur-lex.europa.eu/LexUriServ/LexUriServ.do?uri=OJ:L:2010:276:0033:0079:en:PDF. (2019).

[3] Diaz SL (2018). Conducting and reporting animal experimentation: Quo vadis?. Eur. J. Neurosci..

[4] Smith AJ, Clutton RE, Lilley E, Hansen KEA, Brattelid T (2018). PREPARE: Guidelines for planning animal research and testing. Lab. Anim..

[5] Kilkenny C, Browne WJ, Cuthill IC, Emerson M, Altman DG (2010). Improving bioscience research reporting: the ARRIVE guidelines for reporting animal research. PLoS Biol..

[6] Smith D (2018). Classification and reporting of severity experienced by animals used in scientific procedures: FELASA/ECLAM/ESLAV Working Group report. Lab. Anim..

[7] Morton DB, Griffiths PH (1985). Guidelines on the recognition of pain, distress and discomfort in experimental animals and an hypothesis for assessment. Vet. Rec..

[8] Roughan JV, Flecknell PA (2003). Evaluation of a short duration behaviour-based post-operative pain scoring system in rats. Eur. J. Pain.

[9] Graf R, Cinelli P, Arras M (2016). Morbidity scoring after abdominal surgery. Lab. Anim..

[10] Deacon RM (2006). Burrowing in rodents: A sensitive method for detecting behavioral dysfunction. Nat. Protoc..

[11] Jirkof P (2010). Burrowing behavior as an indicator of post-laparotomy pain in mice. Front. Behav. Neurosci..

[12] Shepherd AJ, Cloud ME, Cao YQ, Mohapatra DP (2018). Deficits in burrowing behaviors are associated with mouse models of neuropathic but not inflammatory pain or migraine. Front. Behav. Neurosci..

[13] Lofgren J (2018). Analgesics promote welfare and sustain tumour growth in orthotopic 4T1 and B16 mouse cancer models. Lab. Anim..

[14] Hohlbaum K (2017). Severity classification of repeated isoflurane anesthesia in C57BL/6JRj mice-assessing the degree of distress. PLoS ONE.

[15] Hager C (2018). Running in the wheel: Defining individual severity levels in mice. PLoS Biol..

[16] Hajian-Tilaki K (2013). Receiver operating characteristic (ROC) curve analysis for medical diagnostic test evaluation. Caspian J. Intern. Med..

[17] Hawkins P (2011). A guide to defining and implementing protocols for the welfare assessment of laboratory animals: Eleventh report of the BVAAWF/FRAME/RSPCA/UFAW Joint Working Group on Refinement. Lab. Anim..

[18] Baumans V (2005). Science-based assessment of animal welfare: Laboratory animals. Rev. Sci. Tech..

[19] Harikrishnan VS, Hansen AK, Abelson KS, Sorensen DB (2018). A comparison of various methods of blood sampling in mice and rats: Effects on animal welfare. Lab. Anim..

[20] Peng M (2016). Battery of behavioral tests in mice to study postoperative delirium. Sci. Rep..

[21] Moore ES (2017). Comparing phlebotomy by tail tip amputation, facial vein puncture, and tail vein incision in C57BL/6 mice by using physiologic and behavioral metrics of pain and distress. J. Am. Assoc. Lab. Anim. Sci..

[22] Hurst JL, West RS (2010). Taming anxiety in laboratory mice. Nat. Methods.

[23] Kim J (2017). Detection of early pancreatic ductal adenocarcinoma with thrombospondin-2 and CA19–9 blood markers. Sci. Transl. Med..

[24] Booken N (2008). Sezary syndrome is a unique cutaneous T-cell lymphoma as identified by an expanded gene signature including diagnostic marker molecules CDO1 and DNM3. Leukemia.

[25] Guthrie E (2003). Cluster analysis of symptoms and health seeking behaviour differentiates subgroups of patients with severe irritable bowel syndrome. Gut.

[26] Giebeler A (2009). c-Met confers protection against chronic liver tissue damage and fibrosis progression after bile duct ligation in mice. Gastroenterology.

[27] Modica S (2012). Selective activation of nuclear bile acid receptor FXR in the intestine protects mice against cholestasis. Gastroenterology.

[28] Ding BS (2014). Divergent angiocrine signals from vascular niche balance liver regeneration and fibrosis. Nature.

[29] Scholten D, Trebicka J, Liedtke C, Weiskirchen R (2015). The carbon tetrachloride model in mice. Lab. Anim..

[30] Cubero FJ (2016). Combined activities of JNK1 and JNK2 in hepatocytes protect against toxic liver injury. Gastroenterology.

[31] Khan N, Kuo A, Brockman DA, Cooper MA, Smith MT (2018). Pharmacological inhibition of the NLRP3 inflammasome as a potential target for multiple sclerosis induced central neuropathic pain. Inflammopharmacology.

[32] Ghallab A (2019). Influence of liver fibrosis on lobular zonation. Cells.

[33] Bleich A, Tolba RH (2017). How can we assess their suffering? German research consortium aims at defining a severity assessment framework for laboratory animals. Lab. Anim..

[34] Ezure T (2000). The development and compensation of biliary cirrhosis in interleukin-6-deficient mice. Am. J. Pathol..

[35] Georgiev P (2008). Characterization of time-related changes after experimental bile duct ligation. Br. J. Surg..

[36] Gabele E (2009). TNFalpha is required for cholestasis-induced liver fibrosis in the mouse. Biochem. Biophys. Res. Commun..

[37] Yi HS (2015). Treatment with 4-methylpyrazole modulated stellate cells and natural killer cells and ameliorated liver fibrosis in mice. PLoS ONE.

[38] Yoshioka H (2017). Vitamin D3-induced hypercalcemia increases carbon tetrachloride-induced hepatotoxicity through elevated oxidative stress in mice. PLoS ONE.

[39] Munafo MR, Davey Smith G (2018). Robust research needs many lines of evidence. Nature.

[40] Habib A (2018). Inhibition of monoacylglycerol lipase, an anti-inflammatory and antifibrogenic strategy in the liver. Gut.

[41] Zhang K (2017). The liver-enriched lnc-LFAR1 promotes liver fibrosis by activating TGFbeta and Notch pathways. Nat. Commun..

[42] Yanguas SC (2016). Experimental models of liver fibrosis. Arch. Toxicol..

[43] Liedtke C (2013). Experimental liver fibrosis research: Update on animal models, legal issues and translational aspects. Fibrogen. Tissue Repair.

[44] Manibusan MK, Odin M, Eastmond DA (2007). Postulated carbon tetrachloride mode of action: A review. J. Environ. Sci. Health C Environ. Carcinog. Ecotoxicol. Rev..

[45] Secklehner J, Richardson CA (2015). The reporting of animal welfare details in liver research: A review of studies describing bile duct ligation in mice (2011–2013). J. Hepatol..

[46] Liles JH, Flecknell PA (1993). The influence of buprenorphine or bupivacaine on the post-operative effects of laparotomy and bile-duct ligation in rats. Lab. Anim..

[47] Jirkof P (2017). Side effects of pain and analgesia in animal experimentation. Lab. Anim. (NY).

[48] Jirkof P (2019). Administration of tramadol or buprenorphine via the drinking water for post-operative analgesia in a mouse-osteotomy model. Sci. Rep..

[49] Ovadia C (2019). Association of adverse perinatal outcomes of intrahepatic cholestasis of pregnancy with biochemical markers: Results of aggregate and individual patient data meta-analyses. Lancet.

[50] Molinaro AM (2015). Diagnostic tests: How to estimate the positive predictive value. Neurooncol. Pract..

[51] Moller C (2018). Toward evidence-based severity assessment in rat models with repeated seizures: I. Electrical kindling. Epilepsia.

[52] Talbot SR (2020). One score to rule them all: severity assessment in laboratory mice. bioRxiv.

[53] Abshagen K (2015). Pathobiochemical signatures of cholestatic liver disease in bile duct ligated mice. BMC Syst. Biol..

[54] Deacon R (2012). Assessing burrowing, nest construction, and hoarding in mice. J. Vis. Exp..

[55] Kumstel S (2019). Grading distress of different animal models for gastrointestinal diseases based on plasma corticosterone kinetics. Animals (Basel).

[56] Bewick V, Cheek L, Ball J (2004). Statistics review 13: Receiver operating characteristic curves. Crit. Care.

[57] R Core Team. The R project for statistical computing. https://www.R-project.org/. (2019).

[58] Kuhn, M. Caret: Classification and regression training. R package version 6.0–84. https://CRAN.R-project.org/package=caret. (2019).

[59] David, M., Evgenia, D., Kurt, H., Andreas, W. & Leisch., F. e1071: Misc functions of the department of statistics, probability theory group (Formerly: E1071), TU Wien. R package version 1.7–2. https://CRAN.R-project.org/package=e1071. (2019).

