# Supplementary material for: Comparing distress of mouse models for liver damage
Source: Sci Rep. 2020 Nov 13;10:19814. doi: 10.1038/s41598-020-76391-w (PMC7666197; doi:10.1038/s41598-020-76391-w)
Supplement: Supplementary file 1 — Supplementary Information [file 41598_2020_76391_MOESM1_ESM.pptx]

## Slide 1
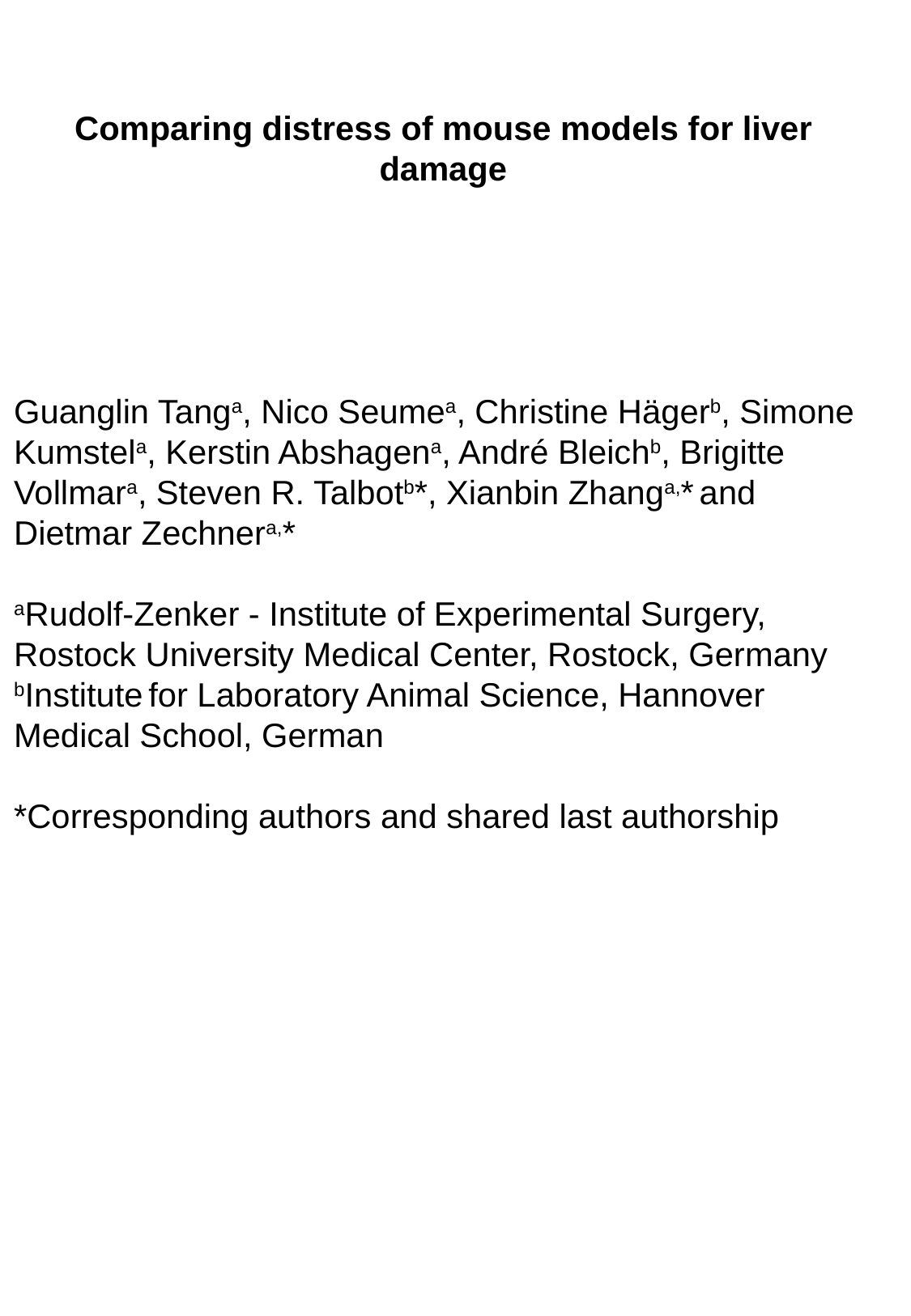

Comparing distress of mouse models for liver damage
Guanglin Tanga, Nico Seumea, Christine Hägerb, Simone Kumstela, Kerstin Abshagena, André Bleichb, Brigitte Vollmara, Steven R. Talbotb*, Xianbin Zhanga,* and Dietmar Zechnera,*
aRudolf-Zenker - Institute of Experimental Surgery, Rostock University Medical Center, Rostock, Germany
bInstitute for Laboratory Animal Science, Hannover Medical School, German
*Corresponding authors and shared last authorship

## Slide 2
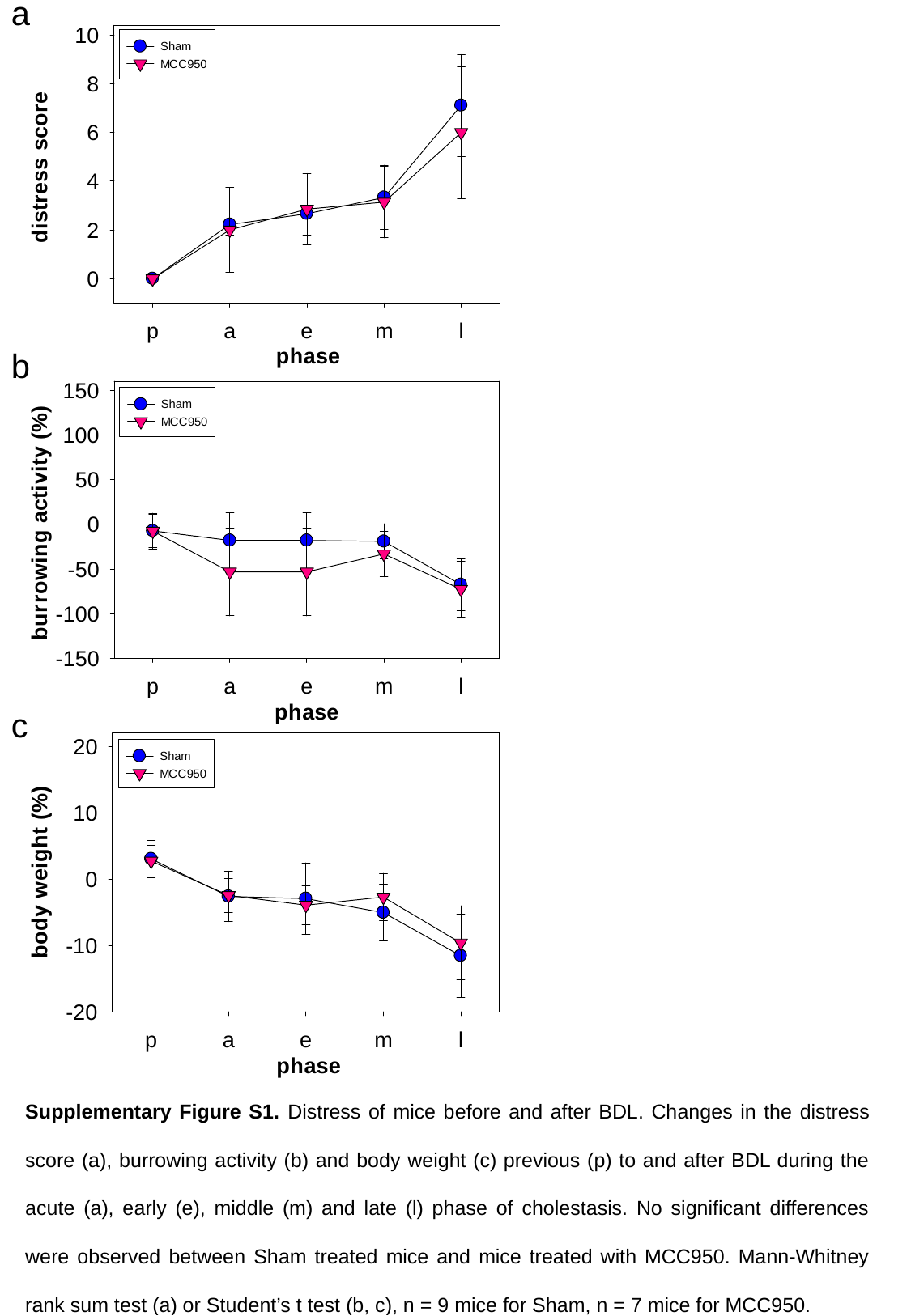

a
b
c
Supplementary Figure S1. Distress of mice before and after BDL. Changes in the distress score (a), burrowing activity (b) and body weight (c) previous (p) to and after BDL during the acute (a), early (e), middle (m) and late (l) phase of cholestasis. No significant differences were observed between Sham treated mice and mice treated with MCC950. Mann-Whitney rank sum test (a) or Student’s t test (b, c), n = 9 mice for Sham, n = 7 mice for MCC950.

## Slide 3
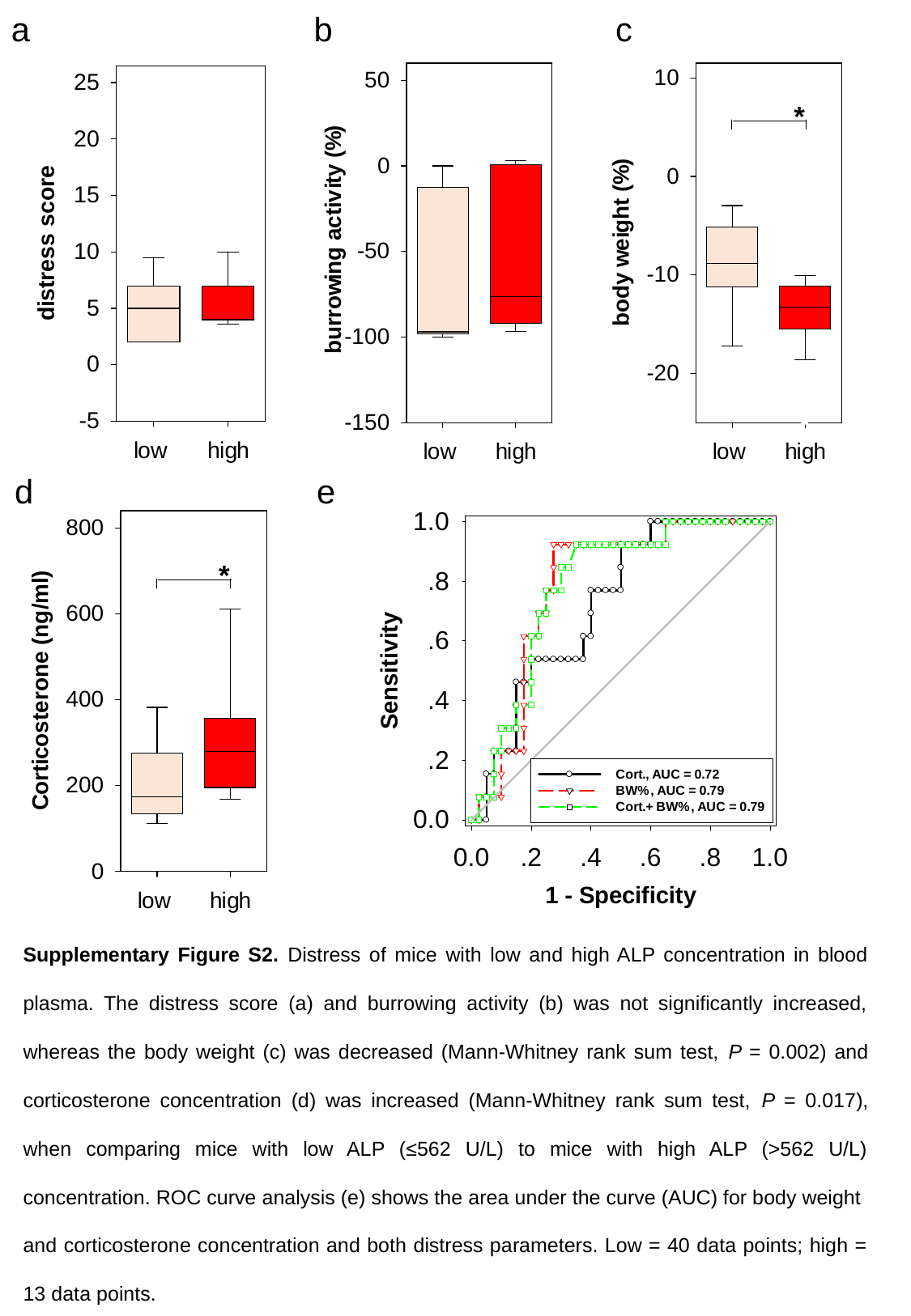

b
c
a
d
Supplementary Figure S2. Distress of mice with low and high ALP concentration in blood plasma. The distress score (a) and burrowing activity (b) was not significantly increased, whereas the body weight (c) was decreased (Mann-Whitney rank sum test, P = 0.002) and corticosterone concentration (d) was increased (Mann-Whitney rank sum test, P = 0.017), when comparing mice with low ALP (≤562 U/L) to mice with high ALP (>562 U/L) concentration. ROC curve analysis (e) shows the area under the curve (AUC) for body weight and corticosterone concentration and both distress parameters. Low = 40 data points; high = 13 data points.
e

## Slide 4
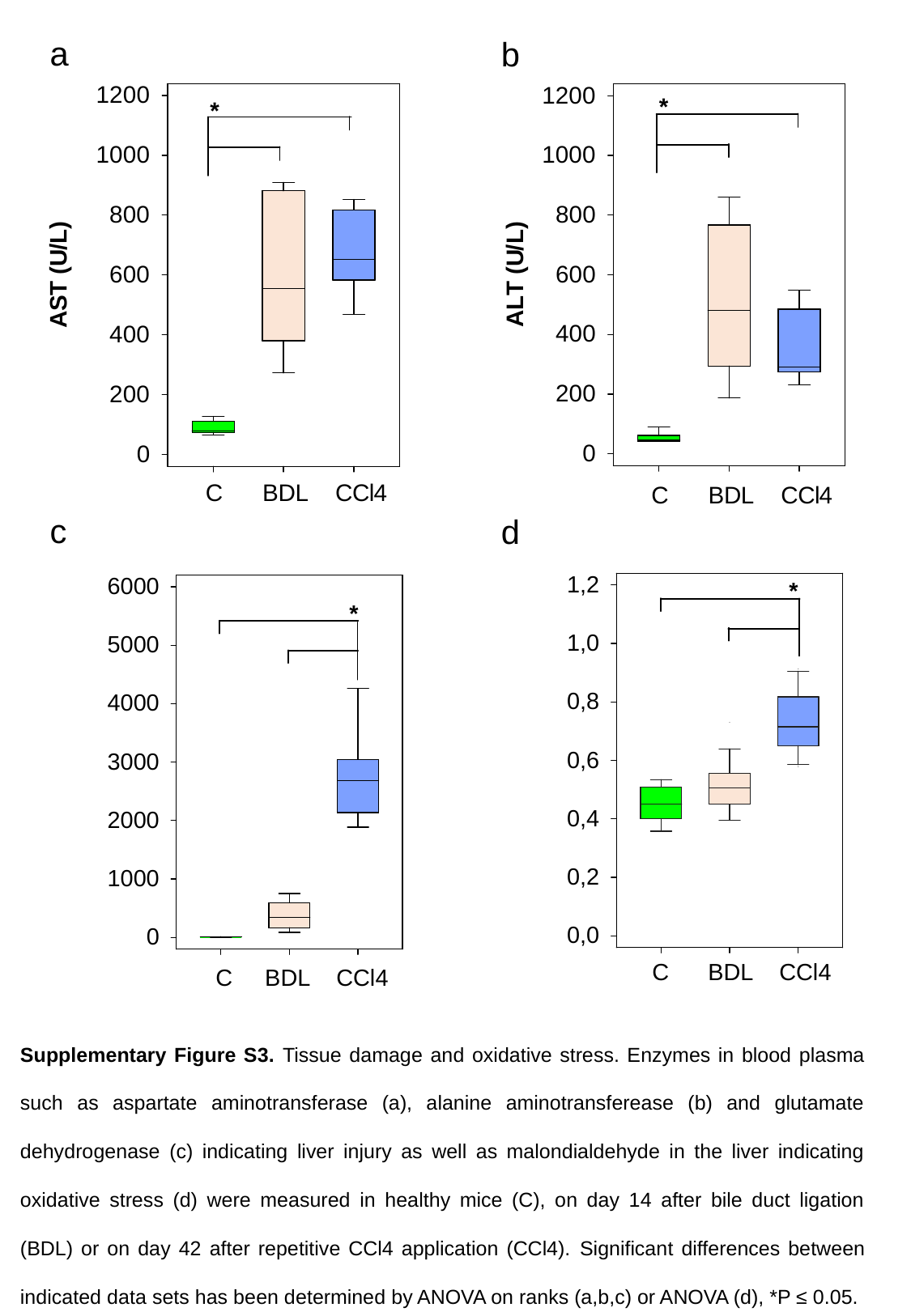

a
b
c
d
Supplementary Figure S3. Tissue damage and oxidative stress. Enzymes in blood plasma such as aspartate aminotransferase (a), alanine aminotransferease (b) and glutamate dehydrogenase (c) indicating liver injury as well as malondialdehyde in the liver indicating oxidative stress (d) were measured in healthy mice (C), on day 14 after bile duct ligation (BDL) or on day 42 after repetitive CCl4 application (CCl4). Significant differences between indicated data sets has been determined by ANOVA on ranks (a,b,c) or ANOVA (d), *P ≤ 0.05.
